# Rapid and accurate taxonomic classification of insect (class Insecta) cytochrome *c* oxidase subunit 1 (COI) DNA barcode sequences using a naïve Bayesian classifier

**DOI:** 10.1111/1755-0998.12240

**Published:** 2014-03-19

**Authors:** Teresita M Porter, Joel F Gibson, Shadi Shokralla, Donald J Baird, G Brian Golding, Mehrdad Hajibabaei

**Affiliations:** * McMaster University, Department of Biology1280 Main Street West, Hamilton, ON, Canada, L8S 4K1; † Biodiversity Institute of Ontario & Department of Integrative Biology, University of Guelph50 Stone Road East, Guelph, ON, Canada, N1G 2W1; ‡ Environment Canada at Canadian Rivers Institute, Department of Biology, University of New BrunswickFredericton, NB, Canada, E3B 6E1

**Keywords:** biomonitoring, cytochrome *c* oxidase subunit 1 (COI), DNA barcoding, insect, naive Bayesian classifier, taxonomic assignment

## Abstract

Current methods to identify unknown insect (class Insecta) cytochrome *c* oxidase (COI barcode) sequences often rely on thresholds of distances that can be difficult to define, sequence similarity cut-offs, or monophyly. Some of the most commonly used metagenomic classification methods do not provide a measure of confidence for the taxonomic assignments they provide. The aim of this study was to use a naïve Bayesian classifier (Wang *et al.* *Applied and Environmental Microbiology*, 2007; 73: 5261) to automate taxonomic assignments for large batches of insect COI sequences such as data obtained from high-throughput environmental sequencing. This method provides rank-flexible taxonomic assignments with an associated bootstrap support value, and it is faster than the blast-based methods commonly used in environmental sequence surveys. We have developed and rigorously tested the performance of three different training sets using leave-one-out cross-validation, two field data sets, and targeted testing of Lepidoptera, Diptera and Mantodea sequences obtained from the Barcode of Life Data system. We found that type I error rates, incorrect taxonomic assignments with a high bootstrap support, were already relatively low but could be lowered further by ensuring that all query taxa are actually present in the reference database. Choosing bootstrap support cut-offs according to query length and summarizing taxonomic assignments to more inclusive ranks can also help to reduce error while retaining the maximum number of assignments. Additionally, we highlight gaps in the taxonomic and geographic representation of insects in public sequence databases that will require further work by taxonomists to improve the quality of assignments generated using any method.

## Introduction

Currently, identification of insects collected from the field using morphological traits is time-consuming and requires specialist knowledge. Identification of adult specimens is often only possible for relatively inclusive taxonomic ranks such as family or order, and the identification of immature or larval specimens is even more challenging. As a result of this, the public databases (GenBank and BOLD) are filled with many insufficiently identified DNA barcode sequences (Kwong *et al.* 2012). We borrow the term ‘insufficiently identified’ from Nilsson *et al.* (2005) to describe sequences that are identified to higher (more inclusive) taxonomic ranks, as opposed to ‘fully identified’ sequences that are identified to the species rank. What is urgently needed is a reference set of DNA barcode sequences from fully identified insects classified to the species rank. Primers and protocols already exist for generating the sequence data (Folmer *et al.* 1994; Hebert *et al.* 2003), and methods have been optimized so that sequences can be generated in a high-throughput manner (Hajibabaei *et al.* 2005). As in many other fields, the main bottleneck in this process is not the production of sequences, but rather the accurate taxonomic identification of samples to the species rank by acknowledged specialists. The quality of the taxonomic identification of samples ultimately determines the usefulness of DNA barcode sequences in the reference databases.

Despite substantial gaps in the reference sequence databases, they are still being used for both automated and manual classifications using tools such as blast (Altschul *et al.* 1997), lowest common ancestor methods (Huson *et al.* 2011), phylogeny-based methods (Munch *et al.* 2008) or kmer-based methods (Wang *et al.* 2007). As a result of the decreasing costs of next-generation sequencing (NGS), many insufficiently identified cytochrome *c* oxidase subunit 1 (COI, CO1, *cox*1, *cox*I) sequences are being generated from metagenomic samples (Damon *et al.* 2010; Hajibabaei *et al.* 2011, 2012; Porter *et al.* 2013). As it is impractical to identify sequences from bulk samples manually, we currently rely on automated sequence comparison tools for classification. NGS platforms produce short sequences or partial ‘mini-barcode’ sequences (Hajibabaei *et al.* 2006) from bulk samples. Previous studies using various regions of ribosomal DNA have shown that decreasing query sequence length and taxonomic assignment rank both affect the accuracy of taxonomic assignments requiring that any new method provides classification accuracy estimates for a range of parameters so that users can implement intelligent experimental designs (Liu *et al.* 2008; Porter & Golding 2011, 2012; Mizrahi-Man *et al.* 2013).

A method that improves confidence for taxonomic assignments is required for large studies to quickly and accurately screen out unreliable assignments. In this context, confidence estimates (bootstrap support values) are provided in an attempt to reduce the rate of erroneous taxonomic assignments. Considering the incomplete nature of current DNA barcode reference databases (Kwong *et al.* 2012; Kvist 2013), automated assignment methods are particularly prone to error due to missing taxa in the reference database.

The purpose of this study was to set up a fast, easy-to-use resource to classify large batches of unknown insect (class Insecta) sequences. We used the naïve Bayesian classifier (NBC or ‘the classifier’) initially developed for classifying bacterial 16S rRNA genes (Wang *et al.* 2007). We created three training sets to classify Insecta COI sequences by mining COI sequences from GenBank. We show that a naïve Bayesian classifier can quickly and accurately generate taxonomic assignments for full- and partial-length insect COI DNA barcode sequences.

## Methods

### Naïve Bayesian classifier

The naïve Bayesian classifier that we used was first developed to classify prokaryote 16S ribosomal RNA sequences (Wang *et al.* 2007). More recently, a training set was developed to use this tool to classify fungal large subunit ribosomal RNA sequences (Liu *et al.* 2012). In this study, we used the Ribosomal Database Project naïve Bayesian classifier version 2.5 available from http://sourceforge.net/projects/rdp-classifier/. Briefly, the classifier is trained using two files: a Fasta formatted sequence file and a text file describing the relationships between taxa at all ranks. For simplicity, we used GenBank taxonomy, and instructions on how to modify this are available from Dryad doi:10.5061/dryad.bc8pc. We describe the details for how we created these training files in the section below. Details for how the classifier works are available in the original publication (Wang *et al.* 2007). Briefly, once trained, the classifier breaks up a query sequence into all possible 8-mer oligos (k-mers). The probability of a query with this particular set of oligos belonging to any of the genera in the training set is calculated. The genus with the highest probability score becomes the taxonomic assignment, and the associated lineage (family, order, class, phylum, kingdom) is retrieved. Statistical support for the assignment is calculated by sampling a subset of 8-mer oligos from the query sequence and repeating the assignment process. Bootstrap confidence estimations are performed by resampling the data 100 times.

### Mining insect sequences from GenBank

The term ‘insect’, as used here, refers to taxa within the class Insecta unless otherwise specified. In GenBank query 1, we used the terms ‘“Insecta”[ORGN] AND “species”[RANK]’ to search the taxonomy database [20 March 2013] to retrieve a list of all insects identified to the species rank using an Ebot script (written by Eric W. Sayers, available from http://www.ncbi.nlm.nih.gov/Class/PowerTools/eutils/ebot/ebot.cgi). We excluded species names if they were not fully identified to the species rank, that is, they contained the terms ‘sp.’, ‘nr.’, ‘aff.’ or ‘cf.’. A second modified query 1 was also used to target insect taxa identified to at least the family rank, and permitted names containing the terms ‘sp.’, ‘aff.’, etc., so as to include as many quality GenBank sequences as possible in our family trained classifier. These two taxon lists were alternatively used in GenBank query 2 ‘ (“cox1”[gene] OR “coxI”[gene] OR “CO1”[gene] OR “COI”[gene]) AND *taxa from query 1*[ORGN]’ to search the nucleotide database [20 March 2013] to retrieve all annotated COI sequences for insects using custom Perl scripts and Bioperl modules (Stajich *et al.* 2002). The additional term ‘AND “barcode”[keyword]’ was appended to GenBank query 2 for another nucleotide database search [25 April 2013]. Sequences shorter than 500 bp, containing missing data (Ns), or containing nucleotide ambiguity codes were excluded. Duplicate sequences for the same species were retained when possible. This was done to improve the representation of sequence variation within species. The GenBank taxonomic lineage (kingdom, phylum, class, order, family, genus, species) for each sequence was retained. Occasionally, classifications to intermediate ranks were missing (not provided to GenBank). These sequences were discarded if the missing rank was at the genus level (for a set trained to the genus rank) or to the family level (for a set trained to the family rank).

### Training and testing the naïve Bayesian classifier

Three training sets were created: (i) a ‘GenBank-genus’ set to train the classifier to make taxonomic assignments to the genus rank, (ii) a ‘GenBank-barcode’ set to train the classifier to make assignments to the genus rank and (iii) a ‘GenBank-family’ set to train the classifier to make assignments to the family rank. The GenBank-genus training set is therefore comprised of all the annotated COI insect sequences (>500 bp, no N's or other nucleotide ambiguities) in GenBank with a species-level identification. The GenBank-barcode training set is comprised of all the annotated COI insect sequences in GenBank with a species-level identification whose record contains the ‘barcode’ keyword. GenBank records with the barcode keyword indicate that these sequences are contributions to the BOLD database and are associated with a sequence chromatogram, pictures and additional metadata. The GenBank-family training set is comprised of all the annotated COI insect sequences in GenBank requiring at least a family-level identification. Custom scripts were used to prepare a Fasta formatted file of reference sequences and a taxonomy text file based on the GenBank taxonomy according to the RDP classifier version 2.5 instructions (Wang *et al.* 2007). Leave-one-out cross-validation (LOOCV) was used to test the accuracy of the classifier, by counting the number of correct assignments at variable taxonomic ranks, using the module provided by the RDP classifier version 2.5. The LOOCV process works by removing a sequence from the training set, making a taxonomic assignment while the reference set does not contain the query, then replacing the sequence before the next assignment is made. This process is repeated for each sequence in the training set. The results simulate making taxonomic assignments when the reference database is potentially incomplete, that is, lacking an exact match to the query sequence. We also made taxonomic assignments by taking each sequence from the training set and classifying each one using the original, complete training set. This simulates making taxonomic assignments when the reference database is known to be complete, that is, contains an exact match to the query sequence. This type of testing can reveal areas where taxonomic coverage of the training set is problematic. Additionally, we performed partial sequence length LOOCV testing using the method provided by the RDP classifier version 2.5. We tested lengths of 50, 100, 200 and 400 bp fragments that were randomly sampled from each full-length query sequence to assess classifier performance of the shorter sequence lengths generated by NGS platforms.

We also assembled three taxonomically defined sets of sequences for testing classifier performance from the International Barcode of Life project (iBOL) data release package 3.75 – v1 available from http://www.boldsystems.org/index.php/datarelease. The sequences in each set were confirmed to be at least 500 bp in length and contained no missing or non-nucleotide characters. We chose three insect orders to focus analyses based on their abundance in the iBOL data set (Fig. S1, Supporting Information) and their popularity in previous barcoding studies. The first set is comprised of 82 sequences from the order Mantodea (mantids) that were identified to the genus rank (Table S1, Supporting Information). These sequences had an average length of 871 bp. The order Mantodea is represented by relatively few sequences in the iBOL data set, is present in the GenBank-genus (*N* = 74) and GenBank-family (*N* = 96) training sets but not in the GenBank-barcode (*N* = 0) training set and was used to test whether sequences not present in a training set would be misclassified or remain unclassified. The second set is comprised of 8647 sequences from the order Lepidoptera (butterflies and moths), and each specimen was identified to the order rank in the iBOL data release. These sequences had an average length of 637 bp. Lepidoptera sequences are widely used in barcoding studies, are the third most abundant order in the iBOL data set (10%) and represent the majority of sequences in the GenBank-genus (50%), GenBank-barcode (81%) and GenBank-family (56%) training sets. We expected the NBC to do a very good job at taxonomically assigning Lepidoptera sequences based on their representativeness in our training sets and based on a previous study that showed North American Lepidoptera species to have limited intraspecific variation (Hebert *et al.* 2010). The third set is comprised of 46 223 sequences from the order Diptera (true flies) that were identified to the order rank in the iBOL data release. These sequences had an average length of 627 bp. Diptera sequences are the most abundant in the iBOL data set (54%), but are less well represented in the GenBank-genus (11%), GenBank-barcode (3%) and GenBank-family (7%) trained classifiers. We expected the GenBank-genus trained classifier to correctly classify many Diptera sequences, but we also expected a significant proportion of misclassified and unclassified sequences based on a previous study that showed Diptera species to have high intraspecific variability (Meier *et al.* 2006).

Finally, we wanted to test the ability of the classifier to work with two field data sets. The first field data set included 1052 COI Sanger sequenced Malaise-caught insects from Insecta (*n* = 993), Arachnida (*n* = 59) and Ellipura (*n* = 44) from Costa Rica with an average length of 310 bp (J. F. Gibson, S. Shokralla, T. M. Porter, I. W. King, S. van Konynenburg, D. H. Janzen, W. Hallwachs & M. Hajibabaei, unpublished). Each of the insects caught was identified to the order rank using morphological characteristics. We then classified sequences using our three trained classifiers and imported the results into megan 4.70.4 for visualization (Huson *et al.* 2011). megan uses a lowest common ancestor (LCA) algorithm to summarize heterogeneous taxonomic information among the best blast hits or NBC taxonomic assignments. We used what we determined to be appropriate bootstrap support cut-offs based on the values shown in Table3, Tables S5 and S6 (Supporting Information). We used the following LCA parameters to process the NBC results: minimum support 1, minimum score (i.e. bootstrap support) 20 for order rank assignments using the GenBank-genus trained classifier, 0 for the GenBank-barcode trained classifier and 60 for the GenBank-family trained classifier (or 0 to process all results), top per cent 100, win score 100, minimum complexity 0.44. We also classified these sequences using blast 2.2.26+ (megablast blastn algorithm) against a local installation of the nucleotide database [15 March 2013] using an e-value cut-off of 1e-10. We processed the blast results using the following megan LCA parameters: minimum support 1, minimum score (i.e. bit score) 100, top per cent 1, win score 0, minimum complexity 0.44.

Although the second field data set will be published elsewhere in full, we provide collection and wet laboratory methods here for context. Benthic (aquatic) samples were collected in July 2010 from the University Woodlot (Corbett Brook) in Fredericton, New Brunswick, Canada. The sample examined in this study was from a conservation area, collected according to Environment Canada's standard benthic macro-invertebrate collection method, a 3-min travelling kick sample (net = 400 *μ*m mesh size), and preserved in 95% ethanol. A small tissue subsample from each of 300 separated individuals was transferred into 96-well plates then subjected to routine analysis following standard DNA barcoding protocols (Folmer *et al.* 1994; Hajibabaei *et al.* 2005; Ratnasingham & Hebert 2007). The remainder of the insect bodies were pooled and divided into multiple MP lysing matrix tubes ‘A’ (100 mg each) and homogenized with the MP FastPrep-24 Instrument (MP Biomedicals Inc.) at speed 6 for 40 s. Total DNA of the slurry was extracted using the NucleoSpin Tissue Kit (Macherey-Nagel Inc.) following the manufacturer's instructions. Two mini-barcode COI fragments were amplified from the bulk sample (130 and 230 bp; Hajibabaei *et al.* 2011) and purified by Qiagen's MinElute PCR purification columns. The amplicons were sequenced on a 454 Genome Sequencer FLX System (Roche Diagnostics GmbH) following the amplicon sequencing protocol. Amplicons were bi-directionally sequenced using 2 (1/16th) regions of a full 70 × 75 PicoTitre sequencing plate. One set of 454-pyrosequencing reads (*N* = 9692) had an average length of 155 bp, and the second set (*N* = 9275) had an average length of 254 bp. Sanger sequences had an average length of 626 bp. The identities of insects were unknown, so the GenBank-genus trained classifier was used to make taxonomic assignments to the family rank.

## Results

### Training set composition

**Fig 1:**
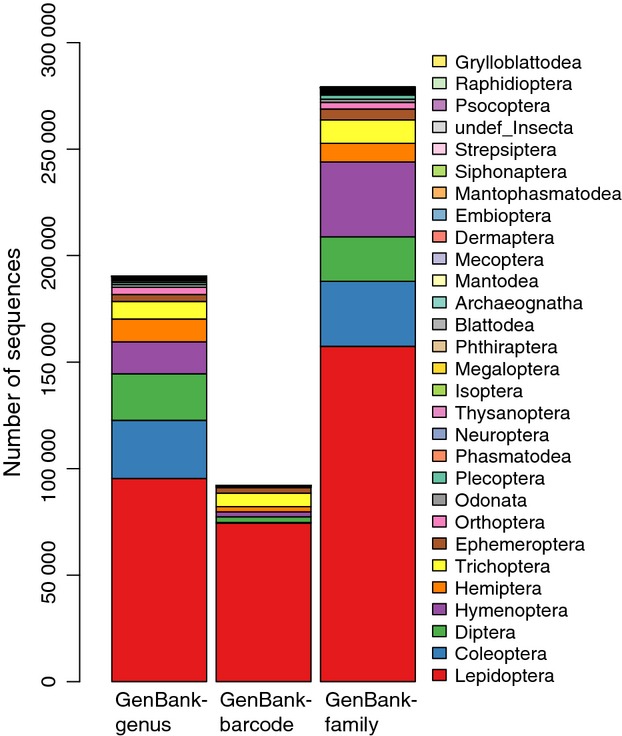
Taxonomic profiles for three insect COI naïve Bayesian classifier training sets. The height of each coloured bar represents the number of sequences from each insect order in three training sets: GenBank-genus, GenBank-barcode and GenBank-family.

Training set composition is summarized in Table1, with the GenBank-genus and GenBank-family sets being the largest and the GenBank-barcode set being the smallest. A breakdown of the total number of unique taxa at each rank is shown in Table S2 (Supporting Information). The GenBank-genus and GenBank-family training sets are represented by 27 370 and 46 815 species, respectively. Note that insufficiently identified species were retained in the GenBank-family training set to increase the diversity of insects represented. Approximately 32% of the genera in the GenBank-genus set, 20% of the genera in the GenBank-barcode set and 13% of the families in the GenBank-family set are comprised of singletons (represented by a single sequence; Table S3, Supporting Information). The taxonomic profile for each training set is summarized in Fig.1 and for each set is dominated by Lepidoptera sequences.

**Table 1:** Composition of three COI insect training sets for the naïve Bayesian classifier

Training set	Trained rank	Number of taxa (all ranks)	Number of sequences
GenBank-genus	Genus	9350	190 333
GenBank-barcode	Genus	3841	92 098
GenBank-family	Family	750	279 130

### LOOCV testing results

**Fig 2:**
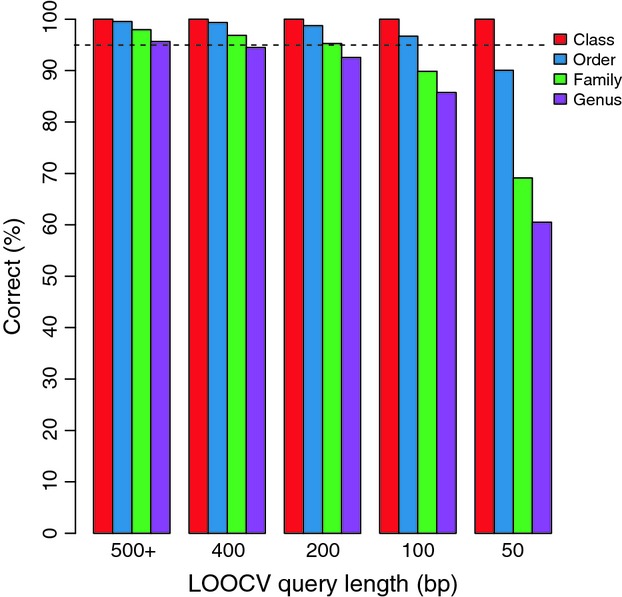
Correctly classified queries during leave-one-out cross-validation of the GenBank-genus trained classifier. No bootstrap support cut-off was used to filter results.

The proportion of correctly classified queries from LOOCV testing, of various lengths, and at various taxonomic ranks is shown in Fig.2 for the GenBank-genus trained classifier. Results for the GenBank-barcode and GenBank-family trained classifiers are shown in Fig S2 and S3 (Supporting Information). For each version of the classifier, the proportion of correct queries was calculated from the ‘total number of correct queries’/‘total number of tested queries’ × 100. Note that the default behaviour of the RDP classifier version 2.5 is that the results for singleton taxonomic assignments are not summarized, thus not shown in these figures. Generally, summarizing taxonomic assignments to more inclusive taxonomic ranks, and using longer query lengths, results in more correctly classified sequences even when no bootstrap support cut-off is applied. Note that the presence of identical sequences for the same genus in the training sets is not expected to affect classification results after the classifier is trained; however, accuracies measured during LOOCV testing may be inflated.

### Reducing taxonomic assignment error

**Fig 3:**
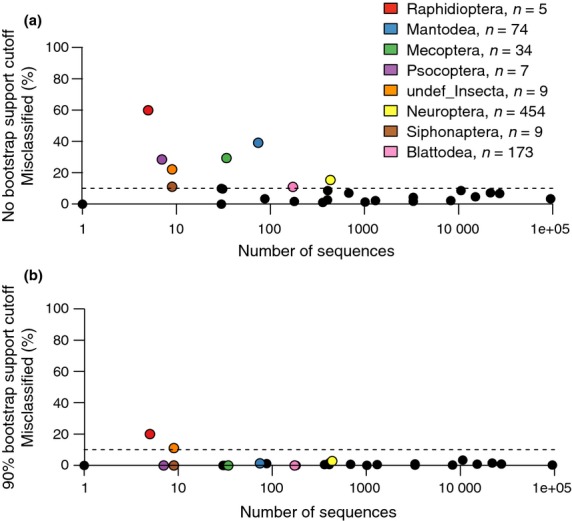
Applying a bootstrap support cut-off reduces the proportion of misclassified sequences. Results from leave-one-out cross-validation of the GenBank-genus trained classifier are shown for each insect order with respect to sample size. Results are shown for misclassified taxa when: (a) no bootstrap support cut-off was used and (b) when a 90% bootstrap support cut-off was used. Points above the dotted line have 10% or greater misclassified sequences. Singletons were included in the calculations.

LOOCV taxonomic assignments from the GenBank-genus trained classifier were analysed without any bootstrap support cut-off and after applying a 90% bootstrap support cut-off to target high confidence assignments (Fig.3). The proportion of misclassified queries per insect order tended to decrease as the number of sequences that represented the order in the reference database increased (Table S4, Supporting Information). Additionally, there was a distinct shift in the proportion of misclassified sequences per order when a 90% bootstrap support cut-off was applied. We noted that two orders, Raphidioptera ‘snakeflies’ and undef_Insecta consistently showed relatively high misclassification rates after LOOCV testing. The category ‘undef_Insecta’ is artificial and is a result of these sequences lacking an order rank classification in GenBank, although many likely belong to the order Zygentoma (silverfish). Applying a minimum bootstrap support cut-off decreased the number of misclassified sequences by 7537 or 4% during LOOCV testing.

Using a potentially incomplete reference database increased rates of type I and type II error as shown in Table2. For all three versions of the classifier, error rates are reduced when a complete reference database is used. Additionally, the rate of type II error (false negatives) tends to be higher than the rate of type I error (false positives). The level of type II error is particularly high for the GenBank-family trained classifier.

**Table 2:** Comparison of type I and type II error rates for three versions of the insect COI classifier used with a complete or potentially incomplete reference database

	Rank	90% bootstrap support cutoff used		
GenBank-genus		GenBank-barcode	GenBank-family
Type I error (%)* Complete reference database	Genus	0.5	0.2	N/A
	Family	0.2	0.0	0.3
	Order	0.1	0.0	0
Type I error (%)† Potentially incomplete reference database	Genus	0.9	0.3	N/A
	Family	0.2	0	0.6
	Order	0.1	0	0.1
Type II error (%)‡ Complete reference database	Genus	1.6	1.0	N/A
	Family	1.1	0.6	11.8
	Order	0.3	0.0	1.9
Type II error (%)§ Potentially incomplete reference database	Genus	2.8	1.8	N/A
	Family	3.1	1.5	15.1
	Order	1.4	0.3	3.6

* Calculated after querying a complete reference database: (total misclassified with 90% bootstrap support or greater (including singletons)/total number of queries) × 100.

† Calculated after querying a potentially incomplete reference database after leave-one-out cross-validation: (total misclassified with 90% bootstrap support or greater (including singletons)/total number of queries) × 100.

‡ Calculated after querying a complete reference database: (total correctly classified with <90% bootstrap support (including singletons)/total number of queries) × 100.

§ Calculated after querying a potentially incomplete reference database after leave-one-out cross-validation: (total correctly classified with <90% bootstrap support/total number of queries) × 100.

In Table3, we provide bootstrap support cut-offs that result in at least 99% of tested queries being correctly classified during LOOCV testing using the GenBank-genus trained classifier. Results for the GenBank-barcode and GenBank-family trained classifiers are shown in Tables S5 and S6 (Supporting Information). Generally, increasing initial query length results in increased classification coverage, even when higher bootstrap support cut-offs are used. Generally, summarizing taxonomic assignments to higher taxonomic ranks enables lower bootstrap support cut-offs to be used, also resulting in the inclusion of a greater number of classifications.

**Table 3:** Bootstrap support cut-offs that result in at least 99% correctly classified queries during leave-one-out cross-validation of the GenBank-genus trained insect COI classifier

Rank	% Bootstrap support cut-off to obtain 99% correctly classified queries*				
	50 bp	100 bp	200 bp	400 bp	FULL (500 bp+)
Genus	N/A	70	70	70	80
Family	N/A	50	40	40	40
Order	95	50	20	0	0

Rank	% Queries classified with the appropriate bootstrap support cut-off (from above)*				
	50 bp	100 bp	200 bp	400 bp	FULL (500 bp+)
Genus	0	55	83	91	93
Family	0	75	93	97	98
Order	28	94	∼100	∼100	∼100

N/A, not available.

Results from singletons not summarized.

### Targeted taxonomic comparisons

In Table4, we compare taxonomic assignments made with the classifier and those made based on morphology-based taxonomic assignments. ‘Matches’ refer to cases where the classifier- and morphology-based assignments match. ‘Nonmatches’ refer to cases where the classifier- and morphology-based assignments do not match. The proportion of Mantodea queries with taxonomic assignments that matched morphology-based assignments was 100% at the order rank for the GenBank-genus and GenBank-family classifiers (Table4). In a situation where there are no exact reference sequences available, as in the GenBank-barcode training set, there are also no correct taxonomic assignments. There are, however, many nonmatch assignments, that is, the Mantodea query sequences were assigned to the next best matching sequences in the training set. Even when representative sequences are missing from the training set, using a sufficiently high bootstrap support cut-off, as well as summarizing assignments to more inclusive taxonomic ranks, could help to avoid making nonmatch taxonomic assignments (Tables S7 and S8, Supporting Information).

**Table 4:** Comparison of order rank taxonomic assignments using three versions of the classifier and morphology-based taxonomic assignments. A ‘match’ is when the classifier and morphology-based taxonomic assignments match. A ‘nonmatch’ is when the classifier and morphology-based taxonomic assignment do not match. ‘Not classified’ refers to when the classifier taxonomic assignment has bootstrap support less than the cut-off value. Bootstrap support cut-offs appropriate for each data set are based on the values from Table3, Tables S5 and S6 (Supporting Information)

Dataset	Taxonomic assignment status	Naïve Bayesian classifier version		
		GenBank-genus	GenBank-barcode	GenBank-family
Mantodea*	Match (%)	82 (100)	0 (0)	82 (100)
	Non-match (%)	0 (0)	82 (100)	0 (0)
	Not classified (%)	0 (0)	0 (0)	0 (0)
Lepidoptera†	Match (%)	8608 (∼100)	8612 (∼100)	8601 (99)
	Non-match (%)	39 (∼0)	35 (∼0)	5 (∼0)
	Not classified (%)	0 (0)	0 (0)	41 (∼1)
Diptera‡	Match (%)	40 636 (88)	33 058 (72)	41 395 (90)
	Non-match (%)	5586 (12)	13 165 (28)	481 (1)
	Not classified (%)	1 (∼0)	0 (0)	4347 (9)
Malaise§	Match (%)	696 (73)	616 (65)	588 (62)
	Non-match (%)	165 (17)	333 (35)	5 (∼1)
	Not classified (%)	88 (9)	0 (0)	356 (38)

* Mantodea (*N* = 82, average length = 871 bp) with bootstrap cut-offs: 0%, 0%, 40% for GenBank-genus, GenBank-barcode and GenBank-family trained classifiers.

† Lepidoptera (*N* = 8647, average length = 637 bp) with bootstrap cut-offs: 0%, 0%, 40%.

‡ Diptera (*N* = 46 223, average length = 627 bp) with bootstrap cut-offs: 0%, 0%, 40%.

§ Malaise (*N* = 949, average length = 310 bp) with bootstrap cut-offs: 20%, 0%, 60%.

For each version of the classifier, 99–100% of Lepidoptera queries had order rank taxonomic assignments that matched the iBOL morphology-based taxonomic assignments (Table4). We expected this high level of taxonomic assignment success because of the well-sampled reference database and relatively low amount of intraspecific variation in Lepidoptera (Hebert *et al.* 2010). There was little advantage to increasing the bootstrap support cut-off values from 0% for Lepidoptera order rank assignments (Table S7, Supporting Information). However, using an 80% bootstrap support cut-off, we show that the GenBank-genus trained classifier can putatively refine 47% and 41% of assignments to the family and genus ranks, respectively (Table S9, Supporting Information). The iBOL Lepidoptera sequences originate from 67 different countries, but the bulk of the sequences represent specimens collected from Canada (32%), Australia (16%) and the United States (16%; Fig. S4, Supporting Information).

For each version of the classifier, 72–90% of Diptera had order rank taxonomic assignments that matched morphology-based assignments (Table4). We expected slightly lower taxonomic assignment success for Diptera compared with Lepidoptera because of the relatively high intraspecific variation previously found in Diptera (Meier *et al.* 2006). The proportion of Diptera queries with order rank taxonomic assignments that do not match the iBOL data is relatively high at 1–28%, and the proportion that remained unclassified ranges from 0 to 9%. When the bootstrap support cut-off is increased and taxonomic assignments are summarized to more inclusive taxonomic ranks, the number of nonmatch assignments was reduced (Tables S7 and S9, Supporting Information). The iBOL Diptera sequences originate from 40 different countries, but the bulk of the sequences represent specimens collected from Canada (80%; Fig. S4, Supporting Information).

### Analysis of insects collected in Malaise traps

We tested the ability of the classifier to handle nontarget query sequences when we used three versions of the classifier with the ‘Insecta+Arachnida+Ellipura’ Malaise data set (*N* = 1052). Note that arthropods in the classes Arachnida and Ellipura were not included in any of our training sets. We determined that it would have been necessary to increase the bootstrap support cut-offs to 45%, 55%, and 55% for the GenBank-genus, GenBank-barcode and GenBank-family trained classifiers, respectively, to reduce the error rate to 0%, assuming that all morphology-based taxonomic identifications to the order rank are correct.

We then processed the Insect only portion of the Malaise trap data set (*N* = 949), and for each version of the classifier, 62–73% of order rank taxonomic assignments matched morphology-based assignments (Table4). The proportion of order rank taxonomic assignment mismatches ranged from ∼1 to 35%, and the proportion of unclassified queries ranged from 0 to 38%. When the bootstrap support cut-off was increased to 79%, the number of nonmatch assignments was reduced to zero for the GenBank-barcode classifier (Tables S7, Supporting Information).

**Fig 4:**
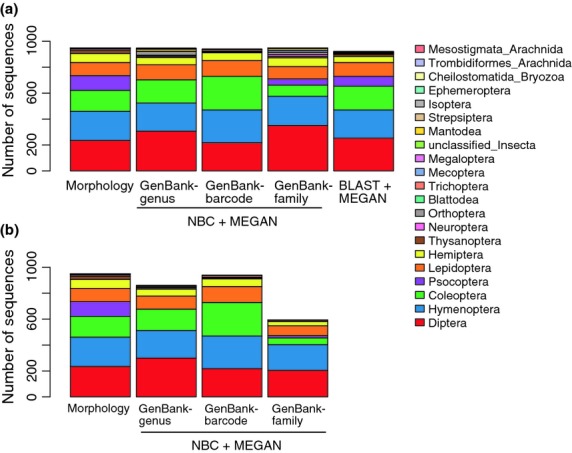
Taxonomic profiles for insects caught in a Malaise trap in Costa Rica. Sequences were classified using morphological characteristics, three versions of the naïve Bayesian classifier (NBC; GenBank-genus, GenBank-barcode, GenBank-family) and blast. Results were imported into megan and taxonomic assignments were summarized at the order rank. Results are shown when: (a) no bootstrap support cut-off was used for NBC assignments and (b) when bootstrap support cut-offs were used for NBC assignments (GenBank-genus 20%, GenBank-barcode 0% and GenBank-family 60%). Bootstrap support cut-offs are chosen from Table3, Tables S5, and S6 (Supporting Information).

**Fig 5:**
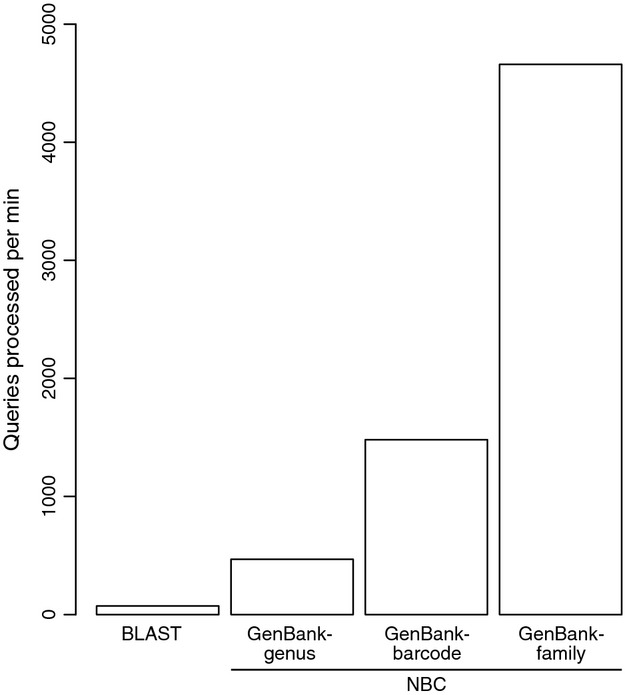
Number of insects from a Malaise trap in Costa Rica taxonomically assigned per minute when using blast or the naïve Bayesian classifier. For each method, 949 ‘Insecta only’ queries were processed and elapsed times recorded. For blast (megablast), the time for a search using a single processor against a local installation of the nucleotide database was recorded. For the naïve Bayesian classifier, the time to make taxonomic assignments and 100 bootstrap support replicates with the GenBank-genus, GenBank-barcode and GenBank-family trained classifiers are recorded.

Diversity profiles of the Insecta only portion of the Malaise data set (*N* = 949) were analysed using the taxonomic assignments provided by morphology, blast and the three versions of the NBC after megan parsing (Fig.4). Assignments were summarized to the order rank. The dominant groups identified using blast and NBC (no bootstrap support cut-off) were similar: Diptera (flies), Hymenoptera (sawflies, wasps, bees, ants), Coleoptera (beetles) and Lepidoptera (moths and butterflies). Another somewhat dominant group is Psocoptera (booklice) represented by 133 insect queries, but only some of these were identified with blast (76), GenBank-genus (2) and the GenBank-family (48) trained classifiers. Psocoptera were not detected at all using the GenBank-barcode training set, but note that Psocoptera were absent from this training set. Applying bootstrap support cut-offs reduced the overall number of taxonomic assignments from the NBCs by 0–38%. Using any version of the NBC, all queries were processed in about 2 min or less, compared with blast searches that took approximately 13 min for this modestly sized data set (*N* = 949; Fig.5).

### Analysis of benthic insect larvae from Corbett Brook

**Fig 6:**
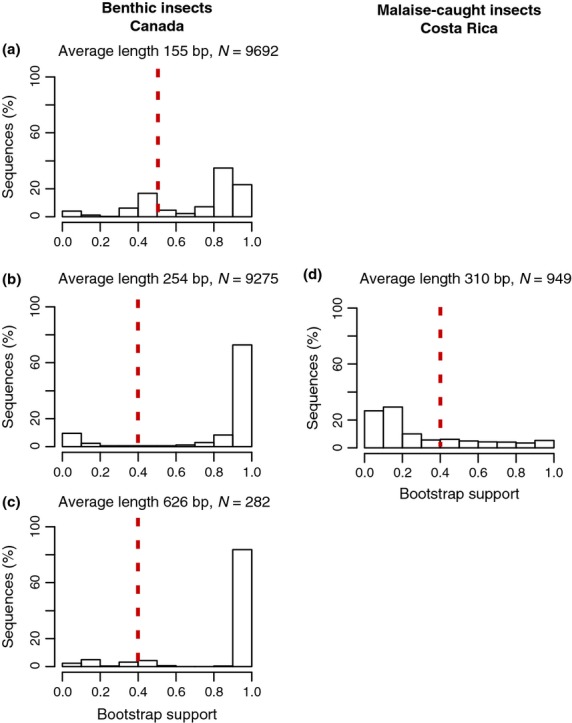
Distribution of naïve Bayesian classifier bootstrap support values for a Canadian benthic insect data set and one Malaise insect data set from Costa Rica. Taxonomic assignments are summarized to the family rank. Bootstrap proportions are pooled into 0.1 (10% bootstrap support) bins, and the proportion of sequences from each data set in each bin is recorded. Data are shown for Canadian benthic insects: (a) 9692 454-pyrosequencing reads with an average length of 155 bp, (b) 9275 454-pyrosequencing reads with an average length of 256 bp and (c) 282 Sanger sequences with an average length of 646 bp, as well as Malaise-caught insects from Costa Rica: (d) 949 Sanger sequences with an average length of 310 bp. Reads are classified using the GenBank-genus trained classifier. The red dotted line shows minimum bootstrap support cut-offs from Table3.

A total of 19 249 sequences were analysed from benthic insects collected from New Brunswick, Canada. These sequences fell into three read length classes according to amplicon size and sequencing method with the following average lengths: 155 bp (454-pyrosequencing), 254 bp (454-pyrosequencing) and 626 bp (Sanger sequencing). As shown in Fig.6, the proportion of reads classified with higher bootstrap support values increases with read length. Nearly 20% more reads are classified with a bootstrap support value greater or equal to 80% when the average query length increases from 155 bp (64%) to 254 bp (84%). The proportion of reads classified with bootstrap support greater or equal to 80% is similar for reads with an average read length of 254 and 626 bp. For comparison, a similar histogram is shown for tropical Malaise-trap-collected insects from Costa Rica. Generally, Canadian benthic insect larvae were classified with higher support values than the tropical Malaise-trap-collected insects from Costa Rica.

## Discussion

### Speed and accuracy of the naïve Bayesian classifier

Previous studies have shown the naïve Bayesian classifier to be both fast and accurate when providing taxonomic assignments for prokaryote 16S ribosomal RNA and fungal large subunit ribosomal RNA genes (Wang *et al.* 2007; Liu *et al.* 2008, 2012; Porter & Golding 2012). In this study, we show that on a 2.2-GHz AMD Opteron 6174 processor, the program classifies and performs 100 bootstrap replicates for approximately 8–78 queries per second for the GenBank-genus and GenBank-family trained classifiers, respectively. Because this method performs significantly faster than blast-based methods, this makes it particularly well suited for quick classification of sequences from high-throughput environmental surveys or biomonitoring studies (such as Baird & Hajibabaei 2012; Wang *et al.* 2013). We demonstrate the accuracy of insect COI taxonomic assignments, to variable taxonomic ranks, during LOOCV testing. We found that the largest GenBank-genus and GenBank-family training sets resulted in NBCs able to make the most automated taxonomic assignments that matched morphology-based assignments. This result is in line with a previous study that recommends using the most diverse training set available (Werner *et al.* 2012). We show that partial-length COI sequences 200 bp or greater can be used to make correct taxonomic assignments to variable taxonomic ranks with accuracy similar to using full-length COI sequences during LOOCV testing. Additionally, we show that Canadian benthic insect larvae (254 bp) are assigned to the family rank with similar statistical support as full-length COI sequences (626 bp). These results are consistent with previous studies working with animal COI mini-barcodes (Meusnier *et al.* 2008; Shokralla *et al.* 2011). Even with these encouraging results with partial COI sequences, we still recommend using the longest possible COI sequences for classification whenever possible to obtain the greatest taxonomic specificity, accuracy and statistical support.

### Described insect diversity vs. database sequence representation

Our training sets contain sequences representing up to 29 insect orders with the Lepidoptera, Hymenoptera, Coleoptera and Diptera orders represented by the most sequences. These orders also contain the greatest number of described insect species (Zhang 2011). Based on LOOCV testing with the GenBank-genus trained classifier, taxa from the orders Strepsiptera, Mecoptera, Mantodea, Zygentoma and Psocoptera resulted in misclassification rates exceeding 10% when no bootstrap support cut-off was enforced. Caution when classifying taxa from these orders is warranted even when a bootstrap support cut-off is applied, because the overall number of sequences representing these orders can be relatively small. A comparison of the number of described extant insect taxa and the number of taxa included in the GenBank-genus training set are shown in Table5. On average, only 65% of family-level taxa and 14% of genus level taxa in the class Insecta are represented in the GenBank-genus training set and, by extension, represented by high-quality annotated COI sequences identified to the species rank in the GenBank nucleotide database. These results are in line with those from a previous study (Kvist 2013) and help to explain, at least in part, why classifying bulk field data for insects remains challenging.

**Table 5:** Summary of class Insecta orders described in the literature and represented in the GenBank-genus training set

Order	Families		Genera		References
	No. extant taxa described	No. taxa included in GenBank-genus training set (% of extant)	No. extant taxa described	No. taxa included in GenBank-genus training set (% of extant)	
Archaeognatha [Microcoryphia]	2	2 (100)	64	9 (14)	Mendes (2002)
Blattodea [incl. Isoptera]	17	12 (71)	738	72 (10)	Beccaloni & Eggleton (2011)
Coleoptera	176	118 (67)	29500	1956 (7)	Slipinski *et al.* (2011)
Dermaptera	11	4 (36)	182	5 (3)	Sakai (1982)
Diptera	158	79 (50)	9323	739 (8)	Pape *et al.* (2011)
Embioptera [Embiidina]	11	6 (55)	84	9 (11)	Miller (2009)
Ephemeroptera	42	21 (50)	405	76 (19)	Barber-James *et al.* (2008)
Grylloblattodea	1	1 (100)	5	1 (20)	Grimaldi & Engel (2005)
Hemiptera	108	83 (77)	?	892 (?)	Cryan & Urban (2012); no estimate for number of genera is available
Hymenoptera	89	53 (60)	8359	728 (9)	Huber (2009)
Lepidoptera	131	110 (84)	15528	3362 (22)	van Nieukerken *et al.* (2011)
Mantodea	14	8 (57)	436	56 (13)	Svenson & Whiting (2009)
Mantophasmatodea	2	2 (100)	10	5 (50)	Eberhard *et al.* (2011)
Mecoptera	9	5 (56)	32	7 (22)	Whiting (2002)
Megaloptera	2	2 (100)	33	5 (15)	Winterton *et al.* (2010)
Neuroptera	16	16 (100)	?	69 (?)	Winterton *et al.* (2010); no estimate for number of genera is available
Odonata	31	13 (42)	642	57 (9)	Kalkman *et al.* (2008)
Orthoptera	40	19 (48)	4418	243 (6)	Ingrisch (2011)
Phasmatodea [Phasmida]	13	9 (69)	454	63 (14)	Brock & Marshall (2011)
Phthiraptera	15	7 (47)	50	10 (20)	Durden & Musser (1994)
Plecoptera	16	11 (69)	286	46 (16)	Fochetti & Tierno de Figueroa (2008)
Psocoptera	40	2 (5)	320	2 (1)	Lienhard & Smithers (2002)
Raphidioptera	2	2 (100)	18	4 (22)	Winterton *et al.* (2010)
Siphonaptera	16	3 (19)	246	6 (2)	Whiting *et al.* (2008)
Strepsiptera	8	4 (50)	?	6 (?)	Kathirithamby (1989); no estimate for number of genera is available
Thysanoptera	9	3 (33)	767	44 (6)	Mound (2011)
Trichoptera	49	35 (71)	601	203 (34)	Holzenthal *et al.* (2011)
Zygentoma	3	3 (100)	117	4 (3)	Mendes (2002)
Total	1031	633 (61)	72 618	8679 (12)	
Average	37	23 (65)	2905	310 (14)	

### Under-sampled reference databases

A lack of representative sequences is a common problem in fungal, microbial and meiofaunal environmental sequencing studies and likely applies to many highly diverse insect communities as well (Creer *et al.* 2010; Virgilio *et al.* 2010; Hibbett *et al.* 2011; Wilson *et al.* 2011; Bergsten *et al.* 2012; Wang *et al.* 2013). Certainly, insufficient barcode sampling across species or populations may impact species assignment success (Meier *et al.* 2008; Lou & Golding 2010, 2012; Yassin *et al.* 2010; Dupuis *et al.* 2012; Wang *et al.* 2013). In fact, simulations lowering taxon coverage in reference databases have been shown to increase identification error for Diptera, Lepidoptera and Hymenoptera data sets (Virgilio *et al.* 2012). Most taxonomic assignment methods, including the naïve Bayesian classifier, are not meant to be used for novelty testing and assume that the appropriate reference sequences are present in the database. One study has developed a method to flag taxa that are not present in a reference database using *ad hoc* distance-based thresholds (Virgilio *et al.* 2012). An alternative approach uses a ‘detector’ to flag sequences that represent taxa not present in the reference data set so that they can be removed prior to making taxonomic assignments (Rosen *et al.* 2011; Lan *et al.* 2012). Both of these methods avoid classifying taxa not actually present in a reference database. We assume that these methods have not yet been widely adopted because they have only been recently developed, and possibly because of the technical difficulties in creating the appropriate sets to train a detector in laboratories that do not have access to bioinformatics expertise.

In spite of the shortcomings of the currently available public databases, we have shown the classifier and our training sets to be robust and conservative when taxonomic assignments are filtered by bootstrap support confidence values and summarized to more inclusive taxonomic ranks when possible. The representativeness and accuracy of our training sets at variable taxonomic ranks are a reflection of the current state of taxonomic annotation and sequence quality in GenBank that will improve over time.

### Geographically biased reference databases

By analysing the metadata associated with iBOL sequences, we noticed that the majority of sequences for some insect orders are derived from a single geographic region, for example, 80% of Diptera sequences are from Canada. The dearth of reference sequences from tropical locales may limit the usefulness of this tool (and others) for automating taxonomic assignments of tropical insects at this time. In such cases, our recommendation is consistent with previous studies and we suggest summarizing taxonomic assignments to more inclusive taxonomic ranks such as order or family (Wang *et al.* 2007; Porter & Golding 2011, 2012; Mizrahi-Man *et al.* 2013). This conservative approach maximizes the number of taxonomic assignments and their accuracy at variable taxonomic ranks, although at the cost of reduced taxonomic resolution.

The impacts of increased geographic scale of DNA barcode sampling on species identification rates vary according to study (Lukhtanov *et al.* 2009; Bergsten *et al.* 2012; Dupuis *et al.* 2012). In our study, testing with a data set comprised of Canadian benthic insects resulted in very good family rank taxonomic assignments, whereas similar testing with a data set of tropical Malaise-caught insects resulted in relatively poor family rank taxonomic assignments. The tropical data set was hyperdiverse with representatives from 11 different arthropod orders (of 14 orders in the original study). Up to 51% of the Malaise trap data set queries in this study could not be reliably taxonomically assigned. We think this is due to an incomplete reference database particularly a lack of representative sequences from tropical locales in our training sets. Our results highlight the need for geographically broad sampling, in particular for insect groups with high rates of intraspecific diversity, to make sound taxonomic assignments (Meier *et al.* 2006; Lou & Golding 2012).

## Conclusions

This study shows that a naïve Bayesian classifier can be effectively applied to classify large numbers of mitochondrial COI barcode sequences from insects. We benchmarked the performance of our training sets using partial-length COI sequences, such as those commonly generated by NGS platforms and provided bootstrap support cut-off guidelines. After thorough testing using LOOCV, targeted taxonomic and field data sets, we have developed the following recommendations: (i) If query taxa are suspected to be largely present in GenBank already, then we recommend using the GenBank-genus trained classifier with bootstrap support cut-offs from Table3 to make the largest number of assignments. Summarizing assignments to more inclusive ranks whenever possible will increase the number of correct taxonomic assignments and the overall number of assignments. (ii) If query taxa are from a tropical locale, or otherwise not likely to be well represented in GenBank already, then we recommend using either the GenBank-genus or GenBank-family trained classifiers combined with bootstrap support cut-offs higher than those recommended in Tables3 and S6. The GenBank-family trained classifier tends to be more conservative than the GenBank-genus trained classifier, generally requiring higher bootstrap support cut-offs, but making fewer incorrect taxonomic assignments at the expense of making fewer overall taxonomic assignments, but it also has the fastest run-time. Again, summarizing assignments to more inclusive taxonomic ranks will reduce the chance of making incorrect assignments. With the continued efforts of entomologists willing to share their sequences in public databases, users will be able to reap the benefits of having high-quality sequences from fully identified specimens to help provide accurate taxonomic assignments using automated tools such as this one. With this in mind, future work will develop new training sets that increase the breadth of taxa, and markers, that can be used with the naïve Bayesian classifier.

## Data Accessibility

The Malaise insect data set is from Gibson *et al.* (2014 Submitted) GenBank Accession nos.KJ435325–KJ436376. Fasta files for the Malaise and trimmed benthic insect data sets are available from Dryad: doi:10.5061/dryad.bc8pc. The GenBank-genus, GenBank-barcode and GenBank-family training sets, along with a Perl script, are also available from Dryad.

## Supporting Information

Fig S1 Abundance of taxonomic orders in the iBOL data release package 3.75 – v1 (*N* = 86 306). Each insect order is followed by the number of sequences represented in the iBOL data set. **Fig. S2** Proportion of correctly classified queries during ‘leave-one-out cross-validation’ (LOOCV) testing of the GenBank-barcode trained classifier. No bootstrap support cut-off was used to filter results. **Fig. S3** Proportion of correctly classified queries during ‘leave-one-out cross-validation’ (LOOCV) testing of the GenBank-family trained classifier. No bootstrap support cut-off was used to filter results. **Fig. S4** Country of origin for sequences in the iBOL data release package 3.75 – v1. The top ten most abundant countries are shown for a) Lepidoptera (*N* = 8647) and b) Diptera (*N* = 46 233). Each country is followed by the number of sequences represented in the iBOL data set. **Table S1** List of Mantodea genera included during testing and their GenBank Accessions nos. **Table S2** Number of unique taxa in three insect COI training sets. **Table S3** Number of taxa represented by a single sequence (singletons) in three insect COI training sets (and proportion of total taxa from Table S2 in parentheses). **Table S4** Proportion of sequences misclassified at the genus rank for each insect order after performing ‘leave-one-out cross-validation’ (LOOCV) testing with the GenBank trained classifier. **Table S5** Bootstrap support cut-offs that result in at least 99% correctly classified queries during leave-one-out cross-validation of the GenBank-barcode trained insect COI classifier. **Table S6** Bootstrap cut-offs that result in at least 99% correctly classified queries during leave-one-out cross-validation of the GenBank-family trained insect COI classifier. **Table S7** Comparison of order rank taxonomic assignments using three versions of the classifier vs. known taxonomic assignments based on morphological characters. **Table S8** Naïve Bayesian classifier automated taxonomic assignments of Mantodea sequences (*N* = 82) verified to match known taxonomic assignments from the iBOL data release package 3.75 - v1 using bootstrap support cut-offs from Tables 3, S5, and S6. **Table S9** Lepidoptera (*N* = 8647) and Diptera (*N* = 46 233) sequences from iBOL data release package 3.75 - v1 that were originally taxonomically assigned to the order rank but are putatively refined to the family and genus ranks using the naïve Bayesian classifier using bootstrap support cut-offs from Table 3, S5, and S6.
