# Supplementary material for: Rapid and accurate taxonomic classification of insect (class Insecta) cytochrome c oxidase subunit 1 (COI) DNA barcode sequences using a naïve Bayesian classifier
Source: Mol Ecol Resour. 2014 Mar 19;14(5):929–42. doi: 10.1111/1755-0998.12240 (PMC4282328; doi:10.1111/1755-0998.12240)
Supplement: Fig S1 — Abundance of taxonomic orders in the iBOL data release package 3.75 – v1 (N = 86 306). Each insect order is followed by the number of sequences represented in the iBOL data set. Fig. S2 Proportion of correctly classified queries during ‘leave-one-out cross-validation’ (LOOCV) testing of the GenBank-barcode trained classifier. No bootstrap support cut-off was used to filter results. Fig. S3 Proportion of correctly classified queries during ‘leave-one-out cross-validation’ (LOOCV) testing of the GenBank-family trained classifier. No bootstrap support cut-off was used to filter results. Fig. S4 Country of origin for sequences in the iBOL data release package 3.75 – v1. The top ten most abundant countries are shown for a) Lepidoptera (N = 8647) and b) Diptera (N = 46 233). Each country is followed by the number of sequences represented in the iBOL data set. Table S1 List of Mantodea genera included during testing and their GenBank Accessions nos. Table S2 Number of unique taxa in three insect COI training sets. Table S3 Number of taxa represented by a single sequence (singletons) in three insect COI training sets (and proportion of total taxa from Table S2 in parentheses). Table S4 Proportion of sequences misclassified at the genus rank for each insect order after performing ‘leave-one-out cross-validation’ (LOOCV) testing with the GenBank trained classifier. Table S5 Bootstrap support cut-offs that result in at least 99% correctly classified queries during leave-one-out cross-validation of the GenBank-barcode trained insect COI classifier. Table S6 Bootstrap cut-offs that result in at least 99% correctly classified queries during leave-one-out cross-validation of the GenBank-family trained insect COI classifier. Table S7 Comparison of order rank taxonomic assignments using three versions of the classifier vs. known taxonomic assignments based on morphological characters. Table S8 Naïve Bayesian classifier automated taxonomic assignments of Mantodea sequences (N = 82) v [file men0014-0929-SD1.pdf]

Supplementary Methods

Customizing the training sets

The format for the training files are described in the original RDP classifier version 2.5 sample data folder that comes with the distribution available from http://sourceforge.net/projects/rdp-classifier/ (Wang *et al.* 2007). For each of our training sets, the two files we used to train the classifier are provided so that they can be used to train the classifier or be modified to suit a user's needs if necessary. Using our files, the classifier can be trained to the genus or family rank using GenBank taxonomy and once the assignment is made to the genus/family rank the associated lineage (family, order, class, phylum, kingdom) is retrieved. As insect taxonomy develops, or users' preferred taxonomic scheme varies, changes to the associated taxonomic lineage for any sequence in the training file can be accommodated. Taxonomic changes can be made directly to the all of the affected Fasta headers in the sequence Fasta file. Sequence additions or deletions can also be made directly to the sequence Fasta file. We provide a Perl script in the supplementary material that can then be used to generate the corresponding taxonomy file used to train the classifier to accommodate the taxonomic changes. Instructions for re-training the classifier are clearly described in the original RDP classifier version 2.5 distribution in the sample data folder 'README' file. For simplicity, the only taxonomic ranks supported are kingdom, phylum, class, order, family, and genus for sets trained to the genus rank; and kingdom, phylum, class, order, and family for sets trained to the family rank.

Supplementary Discussion

Sequence annotation in publically available sequences

The impact of insufficiently identified and mis-annotated sequences that may result in the reduced quality of sequences in public databases has been previously evaluated (Nilsson *et al.* 2006; Bidartondo *et al.* 2008; Costa *et al.* 2012; Kwong *et al.* 2012). The result is that many insufficiently identified sequences cannot be utilized for high-resolution taxonomic assignments to species rank. As previously noted, many iBOL sequences are not present in GenBank (Kwong *et al.* 2012) and many COI sequences in GenBank do not contain the 'barcode' keyword. The result is that our GenBank-barcode training set was the smallest and least representative set. It was useful here for comparison purposes, but should not be used for making taxonomic assignments. Additionally, the iBOL data release package 3.75 v1 does not provide corresponding GenBank numbers so cross-referencing sequences from each database can be problematic. Although taxonomic assignments for insects in the iBOL data release package are limited to the order rank, taxonomic assignments to BINs (barcode index numbers) are provided (Ratnasingham & Hebert 2013). For instances where the BIN unit is not sufficient or appropriate, we have shown that in some cases our insect classifier may be able to refine taxonomic assignments to lower (more specific) ranks.

Single marker bias

Problems using a single arbitrary marker, in this case COI, largely focus on its use as the sole piece of evidence for species delimitation and new species discovery (Cognato

2006). The ‘barcoding gap’ refers to a separation between the within species and among species sequence variability for COI sequences (Meyer & Paulay 2005; Meier *et al.* 2008). It has been suggested that this barcoding gap, sometimes used to explain the inability of a barcode assignment method to correctly assign unknown queries, may actually be an artifact of insufficient sampling (Wiemers & Fiedler 2007). We recognize these issues, but agree with the view that a DNA barcode can still be used as a tool to assign unknown specimens to predefined groups (Nielsen & Matz 2006), in this case, genus or family level taxa defined by NCBI taxonomy. Considering that the classifier was trained only to the genus or the family ranks, and that assignments can be summarized to even more inclusive ranks as needed to obtain a minimum level of bootstrap support, taxonomic assignments made with this tool are relatively conservative compared with a BLAST top hit approach (a commonly used method in environmental sequencing studies). Use of this method in conjunction with other taxonomic assignment methods (such as phylogeny, indicator vector, segregating sites, LCA, or coalescent based approaches), and other genes when available, are not mutually exclusive and is encouraged when there is a need to visualize, corroborate, or test taxonomic assignments flagged to be of special interest (Munch *et al.* 2008a,b; Sirovich *et al.* 2009, 2010; Lou & Golding 2010; Yang & Rannala 2010; Huson *et al.* 2011).

Choosing meaningful bootstrap support cutoffs

It has been recently recognized that choosing a suitable bootstrap support cutoff for taxonomic assignments will vary with query length and assignment rank (Mizrahi-Man *et al.* 2013). The most commonly used cutoff is 80% bootstrap proportion for the

Ribosomal Database Project naïve Bayesian classifier with the 16S training sets (Wang *et al.* 2007). Alternatively, a 50% cutoff has been recommended for 16S query sequences shorter than 250 bp (Liu *et al.* 2008; Claesson *et al.* 2009). In this study, we used LOOCV testing to propose bootstrap support cutoffs appropriate for each trained version of the classifier for a variety of query sequence lengths and taxonomic ranks. Note that these values worked well for query sequences known to be present in the reference dataset. We have shown that unknown insect queries from tropical field studies and poorly represented insect orders remain problematic and may require bootstrap support cutoffs higher than those listed in Table 3 (and Tables S5 and S6) to avoid making incorrect taxonomic assignments.

Impact of sequence error on taxonomic assignments

Previous work has also addressed the impact of very low frequency variants due to sequencing error on the taxonomic assignment of COI barcode sequences (Stoeckle & Kerr 2012). A previous study has suggested that the naïve Bayesian classifier that uses k-mer frequencies for classification may be particularly sensitive to sequence error compared with BLAST that uses local sequence alignments (Porter & Golding 2012). This makes sequence quality filtering of sequences especially important prior to using this tool.

Supplementary References

Bidartondo MI, Bruns TD, Blackwell M *et al.* (2008) Preserving accuracy in GenBank. *Science*, **319**, 1616.

Claesson MJ, O'Sullivan O, Wang Q *et al.* (2009) Comparative analysis of pyrosequencing and a phylogenetic microarray for exploring microbial community structures in the human distal intestine. *PLoS ONE*, **4**, e6669.

Cognato AI (2006) Standard percent DNA sequence difference for insects does not predict species boundaries. *Journal of Economic Entomology*, **99**, 1037-1045.

Costa FO, Landi M, Martins R *et al.* (2012) A ranking system for reference libraries of DNA barcodes: application to marine fish species from Portugal. *PLoS ONE*, **7**, e35858.

Huson DH, Mitra S, Ruscheweyh H-J *et al.* (2011) Integrative analysis of environmental sequences using MEGAN4. *Genome Research*, **21**, 1552-1560.

Liu Z, DeSantis TZ, Andersen GL *et al.* (2008) Accurate taxonomy assignments from 16S rRNA sequences produced by highly parallel pyrosequencers. (2008) *Nucleic Acids Research*, **36**, e120.

Lou M, Golding GB (2010) Assigning sequences to species in the absence of large interspecific differences. *Molecular Phylogenetics and Evolution*, **56**, 187-194.

Meier R, Zhang G, Ali F (2008) The use of mean instead of smallest interspecific distances exaggerates the size of the "barcoding gap" and leads to misidentification. *Systematic Biology*, **57**, 809-813.

Meyer CP, Paulay G (2005) DNA barcoding: error rates based on comprehensive sampling. *PLoS Biology*, **3**, e422.

Mizrahi-Man O, Davenport ER, Gilad Y (2013) Taxonomic classification of Bacterial 16S rRNA genes using short sequencing reads: Evaluation of effective study designs. *PLoS ONE*, **8**, e53608.

Munch K, Boomsma W, Huelsenbeck JP et al. (2008a) Statistical assignment of DNA sequences using Bayesian phylogenetics. *Systematic Biology*, **57**, 750-757.

Munch K, Boomsma W, Willerslev E *et al.* (2008b) Fast phylogenetic DNA barcoding. *Philosophical Transactions of the Royal Society B*, **363**, 3997-4002.

Nielsen R, Matz M (2006) Statistical approaches for DNA barcoding. *Systematic Biology*, **55**, 162-169.

Nilsson RH, Ryberg M, Kristiansson E *et al.* (2006) Taxonomic reliability of DNA sequences in public sequence databases: a fungal perspective. *PLoS ONE*, **1**, e59.

Porter TM, Golding GB (2012) Factors that affect large subunit ribosomal DNA amplicon sequencing studies of fungal communities: classification method, primer choice, and error. *PLoS ONE*, **7**, e35749.

Ratnasingham S, Hebert PDN (2013) A DNA-based registry for all animal species: the barcode index number (BIN) system. *PLoS ONE*, **8**, e66213.

Sirovich L, Stoeckle MY, Zhang Y (2009) A scalable method for analysis and display of DNA sequences. *PLoS ONE*, **4**, e7051.

Sirovich L, Stoeckle MY, Zhang Y (2010) Structural analysis of biodiversity. *PLoS ONE*, **5**, e9266.

Stoeckle MY, Kerr KCR (2012) Frequency matrix approach demonstrates high sequence quality in avian BARCODEs and highlights cryptic pseudogenes. PLoS ONE, 7, e43992.

Wang Q, Garrity GM, Tiedje JM *et al.* (2007) Naïve Bayesian classifier for rapid assignment of rRNA sequences into the new bacterial taxonomy. *Applied and Environmental Microbiology*, **73**, 5261-5267.

Wiemers M, Fiedler K (2007) Does the DNA barcoding gap exist? – a case study in blue butterflies (Lepidoptera: Lycaenidae). *Frontiers in Zoology*, **4**, 8.

Yang Z, Rannala B (2010) Bayesian species delimitation using multilocus sequencing data. *Proceedings of the National Academy of Sciences*, **107**, 9264-9269.

Supporting Figure Legends

Figure S1 Abundance of taxonomic orders in the iBOL data release package 3.75 – v1 (N=86,306). Each insect order is followed by the number of sequences represented in the iBOL dataset.

Figure S2 Proportion of correctly classified queries during 'leave one out cross validation' (LOOCV) testing of the GenBank-barcode trained classifier. No bootstrap support cutoff was used to filter results.

Figure S3 Proportion of correctly classified queries during 'leave one out cross validation' (LOOCV) testing of the GenBank-family trained classifier. No bootstrap support cutoff was used to filter results.

Figure S4 Country of origin for sequences in the iBOL data release package 3.75 – v1. The top ten most abundant countries are shown for a) Lepidoptera (N=8,647) and b) Diptera (N=46,233). Each country is followed by the number of sequences represented in the iBOL dataset.

Figure S1

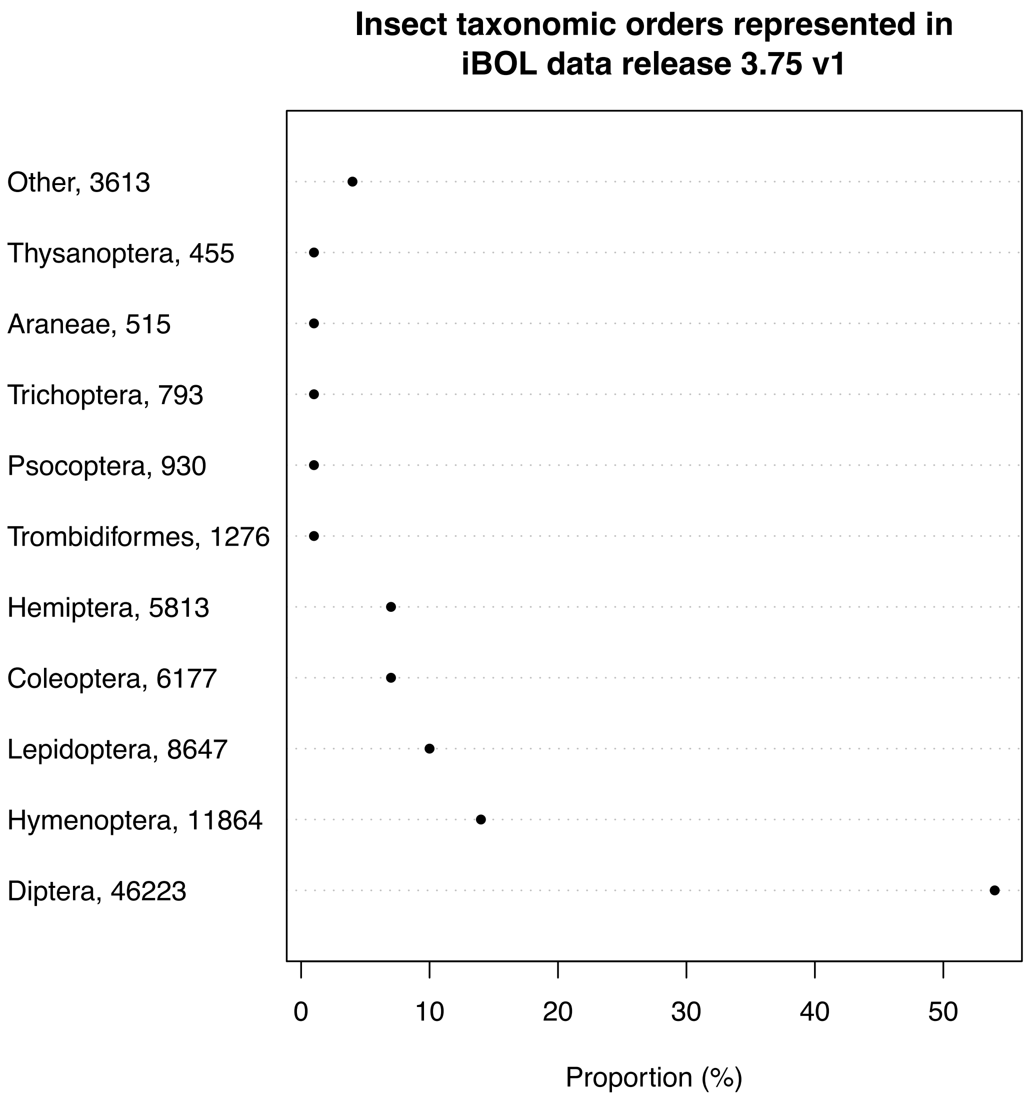
Insect taxonomic orders represented in
iBOL data release 3.75 v1
Other, 3613
Thysanoptera, 455
Araneae, 515
Trichoptera, 793
Psocoptera, 930
Trombidiformes, 1276
Hemiptera, 5813
Coleoptera, 6177
Lepidoptera, 8647
Hymenoptera, 11864
Diptera, 46223
0
10
20
30
40
50
Proportion (%)

Figure S2

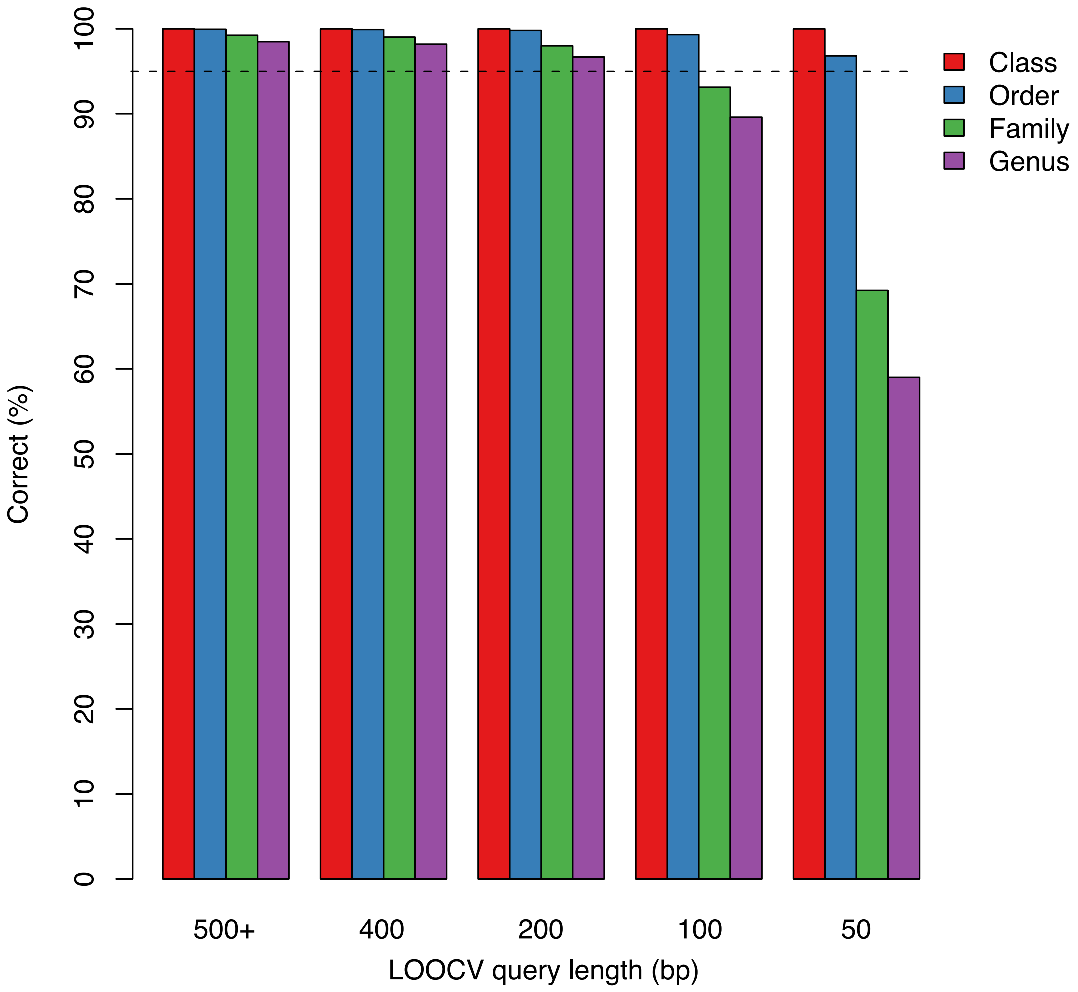

Correct (%)
0
10
20
30
40
50
60
70
80
90
100
500+
400
200
100
50
LOOCV query length (bp)
Class
Order
Family
Genus

Figure S3

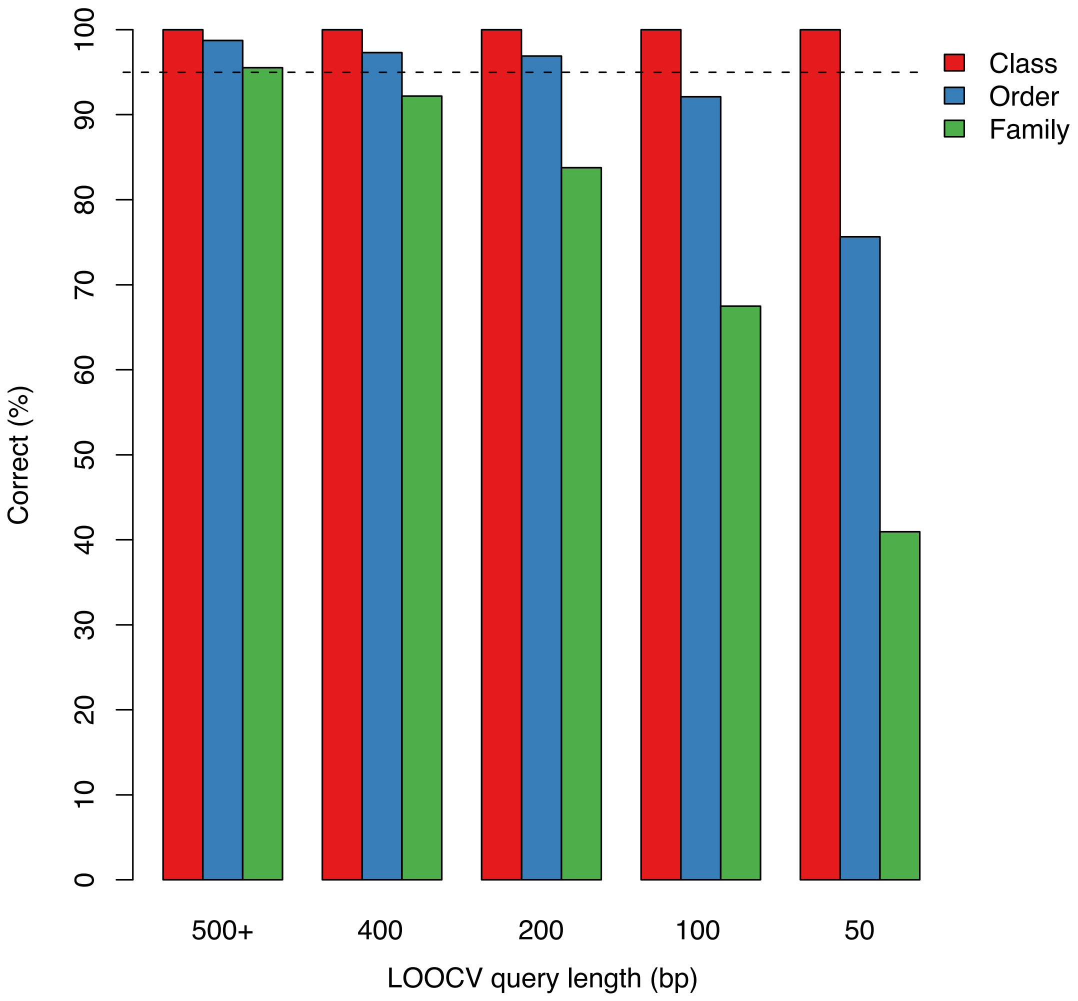
Correct (%)
0
10
20
30
40
50
60
70
80
90
100
Class
Order
Family
500+
400
200
100
50
LOOCV query length (bp)

Figure S4

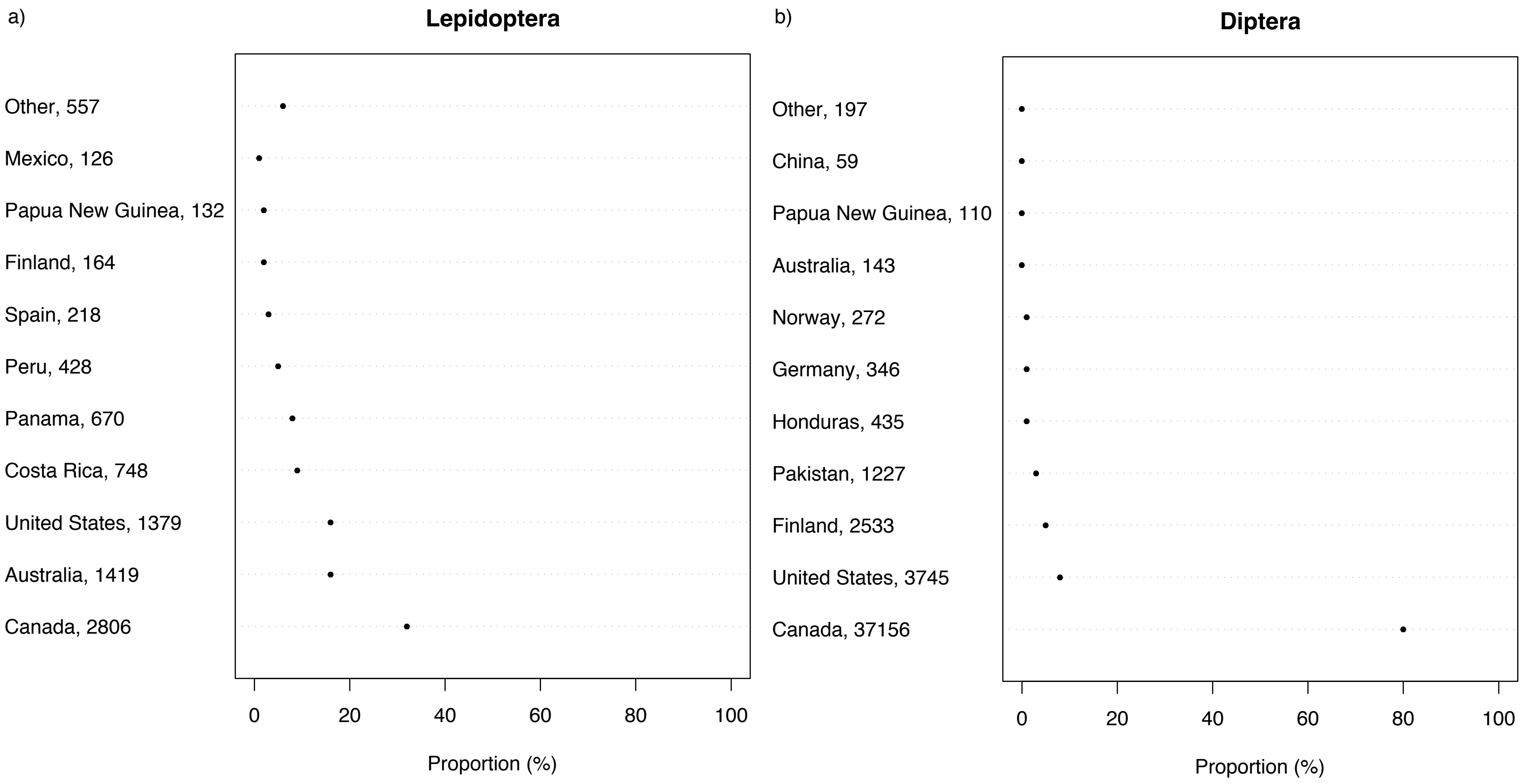
a)
Lepidoptera
Other, 557
Mexico, 126
Papua New Guinea, 132
Finland, 164
Spain, 218
Peru, 428
Panama, 670
Costa Rica, 748
United States, 1379
Australia, 1419
Canada, 2806
0
20
40
60
80
100
Proportion (%)
b)
Diptera
Other, 197
China, 59
Papua New Guinea, 110
Australia, 143
Norway, 272
Germany, 346
Honduras, 435
Pakistan, 1227
Finland, 2533
United States, 3745
Canada, 37156
0
20
40
60
80
100
Proportion (%)

Table S1 – List of Mantodea genera included during testing and their GenBank accessions.

Family	Genus	GenBank Accession
Acanthopidae	Raptrix	FJ802836
Amorphoscelidae	Cliomantis	FJ802777
	Gyromantis	FJ802776
	Paraoxypilus	FJ802755
Empusidae	Empusa	FJ802806
Hymenopodidae	Acromantis	FJ802908
	Amorphoscelis	FJ802783
		FJ802888
		FJ802906
		FJ802821
		FJ802843
	Ceratomantis	FJ802896
	Creobroter	FJ802787
	Ephippiomantis	FJ802899
	Euantissa	FJ802834
	Hestiasula	FJ802909
	Oxypilus	FJ802815
		FJ802845
		FJ802862
		FJ802819
Iridopterygidae	Bolbena	FJ802852
	Hapalomantis	FJ802858
	Hapalopeza	FJ802886
	Miromantis	FJ802907
	Tropidomantis	FJ802910
		FJ802838
		FJ802792
	Xanthomantis	FJ802904
Liturgusidae	Liturgusa	FJ802797
	Stenomantis	FJ802770
Mantidae	Bolbe	FJ802771
	Bolbe	FJ802759
	Coptopteryx	FJ802778
	Deroplatys	FJ802917
		FJ802918
		FJ802895
	Elmantis	FJ802853

Family	Genus	GenBank Accession
		FJ802848
	Entella	FJ802824
	Euchomenella	FJ802786
	Gimantis	FJ802901
	Gonypeta	FJ802894
	Gonypetyllis	FJ802785
	Ligaria	FJ802809
	Ligariella	FJ802772
	Mantis	FJ802846
	Miomantis	FJ802844
		FJ802829
		FJ802823
		FJ802831
	Myrcinus	FJ802851
	Orthoderella	FJ802804
	Parastagmatoptera	FJ802762
	Phyllovates	FJ802839
	Polyspilota	FJ802847
	Pseudomantis	FJ802884
	Rhombodera	FJ802913
	Sphodromantis	FJ802756
	Stagmomantis	FJ802856
		FJ802857
		FJ802761
		FJ802813
		FJ802760
	Statilia	FJ802774
		FJ802781
		FJ802915
		FJ802849
	Tamolanica	NC_007702
	Vates	FJ802803
		FJ802798
		FJ802799
Mantoididae	Mantoida	FJ802822
		FJ802793
		FJ802794
Sibyllidae	Sibylla	FJ802808
Tarachodidae	Dysaules	FJ802835
	Leptomantella	FJ802832

Family	Genus	GenBank Accession
	Pyrgomantis	FJ802825
	Tarachodes	FJ802842
Thespidae	Hoplocorypha	FJ802767
	Musoniella	FJ802800
	Oligonicella	FJ802775

Table S2 Number of unique taxa in three insect COI training sets. The number of species included in the training sets is shown although the classifier was only trained to the genus or family ranks.

**Rank**	**GenBank**	**GenBank-barcode**	**GenBank-family**
Kingdom	1	1	1
Phylum	1	1	1
Class	1	1	1
Order	29	16	29
Family	637	256	718
Genus	8,679	3,565	10,242
Species	27,370	9,991	46,815*

*includes insufficiently identified species assignments including the terms sp., aff., cf., etc.

Table S3 Number of taxa represented by a single sequence (singletons) in three insect COI training sets (and proportion of total taxa from Table S2 in parentheses).

**Rank**	**GenBank**	**GenBank-barcode**	**GenBank-family**
Kingdom	0	0	0
Phylum	0	0	0
Class	0	0	0
Order	1	1	0
Family	76	46	90 (13%)
Genus	2808 (32%)	699 (20%)	N/A

N/A - not applicable since the GenBank-family training set was trained to the family rank

Table S4 Proportion of sequences misclassified at the genus rank for each insect order after performing ‘leave one out cross validation’ (LOOCV) testing with the Genbank trained classifier.

	**GenBank**	
**Insect Orders**	**Number of queries tested**	**% Misclassified[1]**
Archaeognatha	84	0.0
Blattodea	150	5.3
Coleoptera	26,455	6.1
Dermaptera	30	6.7
Diptera	21,538	6.7
Embioptera	24	0.0
Ephemeroptera	3,281	1.6
Grylloblattodea	0	-
Hemiptera	10,230	8.0
Hymenoptera	14,801	4.5
Isoptera	386	2.1
Lepidoptera	94,750	3.2
Mantodea	29	27.6
Mantophasmatodea	30	0.0
Mecoptera	32	28.1
Megaloptera	358	0.6
Neuroptera	389	9.3
Odonata	1,288	1.7
Orthoptera	3,217	3.7
Phasmatodea	649	4.5
Phthiraptera	179	1.7
Plecoptera	1,012	1.1
Psocoptera	6	16.7
Raphidioptera	2	0.0
Siphonaptera	6	0.0
Strepsiptera	5	40.0
Thysanoptera	385	8.1
Trichoptera	8,200	1.9
undef_Insecta	9	22.2

*Taxonomic assignments were not filtered by any bootstrap support cutoff. Results for singletons not shown.

[1](Total misclassified sequences / total tested queries) x 100

'-' Order represented by a single sequence (singleton) so LOOCV result was not summarized

Table S5 Bootstrap support cutoffs that result in at least 99% correctly classified queries during leave-one-out cross-validation of the GenBank-barcode trained insect COI classifier.

	**% Bootstrap support cutoff required to obtain 99% correct classifications**				
**Rank**	**50 bp**	**100 bp**	**200 bp**	**400 bp**	**FULL (500 bp+)**
Genus	N/A	50	30	20	20
Family	95	50	20	0	0
Order	80	0	0	0	0
	**% Queries classified at the appropriate bootstrap support cutoff (from above)**				
Genus	0%	68%	95%	98%	99%
Family	4%	79%	99%	~100%	~100%
Order	85%	~100%	~100%	~100%	~100%

*Results for singletons are not summarized.

N/A = Not available.

Table S6 Bootstrap cutoffs that result in at least 99% correctly classified queries during leave-one-out cross-validation of the GenBank-family trained insect COI classifier.

	**% Bootstrap support cutoff required to obtain 99% correct classifications**				
**Rank**	**50 bp**	**100 bp**	**200 bp**	**400 bp**	**FULL (500 bp+)**
Family	N/A	80	80	80	80
Order	80	60	60	70	40
	**% Queries classified with the appropriate bootstrap support cutoff (from above)**				
Family	0%	20%	47%	76%	89%
Order	17%	74%	95%	96%	99%

*Results for singletons are not summarized.

N/A = Not available.

Table S7 Comparison of order rank taxonomic assignments using three versions of the classifier versus known taxonomic assignments based on morphological characters. Bootstrap support cutoffs were chosen to minimize the number of taxonomic assignments that did not match (Non-match category below) known taxonomic assignments for the classifier with the smallest training set (GenBank-barcode). This table is directly comparable to Table 4.

		**Naïve Bayesian classifier version:**		
**Dataset**		**GenBank**	**GenBank-barcode**	**GenBank-family**
Mantodea[1]				
	Match	79 (96%)	0 (0%)	81 (99%)
	Non-match	0 (0%)	0 (0%)	0 (0%)
	Not classified	3 (4%)	82 (100%)	1 (1%)
Lepidoptera[2]				
	Match	8,605 (~100%)	8,608 (~100%)	8,554 (99%)
	Non-match	2 (~0%)	0 (0%)	4 (~0%)
	Not classified	40 (~0%)	39 (~0%)	89 (1%)
Diptera[3]				
	Match	24,115 (52%)	22,125 (48%)	34,208 (74%)
	Non-match	5 (~0%)	0 (0%)	6 (~0%)
	Not classified	22,103 (48%)	24,098 (52%)	12,009 (26%)
Malaise[4]				
	Match	297 (31%)	225 (24%)	443 (47%)
	Non-match	2 (~0%)	0 (0%)	2 (~0%)
	Not classified	650 (68%)	724 (76%)	504 (53%)

[1]Mantodea (N=82, average length = 871 bp) with bootstrap cutoffs: 96%, 96%, 96% for GenBank, GenBank-barcode, and GenBank-family trained classifiers
[2]Lepidoptera (N=8,647, average length = 637 bp) with bootstrap cutoffs: 60%, 60%, 60%
[3]Didptera (N=46,223, average length = 627 bp) with bootstrap cutoffs: 83%, 83%, 83%
[4]Malaise (N=949, average length = 310 bp) with bootstrap cutoffs: 79%, 79%, 79%

Table S8 Naïve Bayesian classifier automated taxonomic assignments of Mantodea sequences (N=82) verified to match known taxonomic assignments from the iBOL data release package 3.75 - v1 using bootstrap support cutoffs from Tables 3, S5, and S6.

	**GenBank (80% cutoff)**			**GenBank-barcode (20% cutoff)**			**GenBank-family (80% cutoff)**		
	**Order**	**Family**	**Genus**	**Order**	**Family**	**Genus**	**Order**	**Family**	**Genus**
Match	82 (100%)	61 (74%)	54 (66%)	0 (0%)	0 (0%)	0 (0%)	82 (100%)	42 (51%)	N/A
Non-match	0 (0%)	3 (4%)	4 (5%)	82 (100%)	82 (100%)	81 (99%)	0 (0%)	28 (34%)	N/A
Not classified	0 (0%)	18 (22%)	24 (29%)	0 (0%)	0 (0%)	1 (1%)	0 (0%)	12 (15%)	N/A

N/A = Not applicable.

Table S9 Lepidoptera (N=8,647) and Diptera (N=46,233) sequences from iBOL data release package 3.75 - v1 that were originally taxonomically assigned to the order rank but are putatively refined to the family and genus ranks using the naïve Bayesian classifier using bootstrap support cutoffs from Table 3, S5, and S6.

	**GenBank (80% cutoff)**		**GenBank-barcode (20% cutoff)**		**GenBank-family (80% cutoff)**	
**Lepidoptera**	**Family**	**Genus**	**Family**	**Genus**	**Family**	**Genus**
	4,079 (47%)	3,511 (41%)	7,296 (84%)	5,284 (61%)	5,158 (60%)	N/A
**Diptera**						
	10,348 (22%)	7,748 (17%)	22,807 (49%)	19,546 (42%)	17,462 (38%)	N/A

N/A = Not applicable.
